# Influence of drug and polymer molecular weight on release kinetics from HEMA and HPMA hydrogels

**DOI:** 10.1038/s41598-023-42923-3

**Published:** 2023-10-04

**Authors:** Parker Toews, Jeffrey Bates

**Affiliations:** Department of Materials Science and Engineering, University of Utah, 122 Central Campus Drive, Room 304, Salt Lake City, UT 84112 USA

**Keywords:** Gels and hydrogels, Polymer synthesis

## Abstract

Drug release kinetics in two compositions of methacrylate hydrogels were monitored as a function of the hydrogel and drug molecular weight. Through modifying the molecular weight of hydrogels, it was demonstrated how the release could be tuned, allowing for increased stability of hydrogels and enhanced release performance. Spectroscopy techniques such as FTIR and UV–Vis–NIR provided inferences into the chemical structure, target molecule concentration, and optical performance of the studied hydrogels. By studying the 30-day target molecule loading stability of the hydrogels, a relationship between the drug and hydrogel molecular weight, and the drug release kinetics could be determined.

## Introduction

Ophthalmic conditions such as glaucoma, dry-eye, and cataracts impact a substantial part of the global population with 79.6 million, 344 million, and 150 million respectively impacted by these conditions in one way or another either through visual impairment or complete blindness^1–3^. Left untreated, these conditions often have an end result of blindness, which correlates to higher medical expenditures, more care days required, and loss of life quality^4^. Commonly, these conditions are treated, or at least stabilized, through the administration of eye drops. Often these have their own issues with low patient adherence, improper administration, or incorrect dosage to the ophthalmic surface^5^. It is due to this factor that a new solution is desired. With over 125 million contact lens wearers globally in 2018, the use of a similar platform for ophthalmic drug delivery is viable from a user standpoint^6^. Hydrogels have recently been of scientific interest due to their capability to be loaded with medication to allow for drug release localized to the eye^7^.

**Figure 1:**
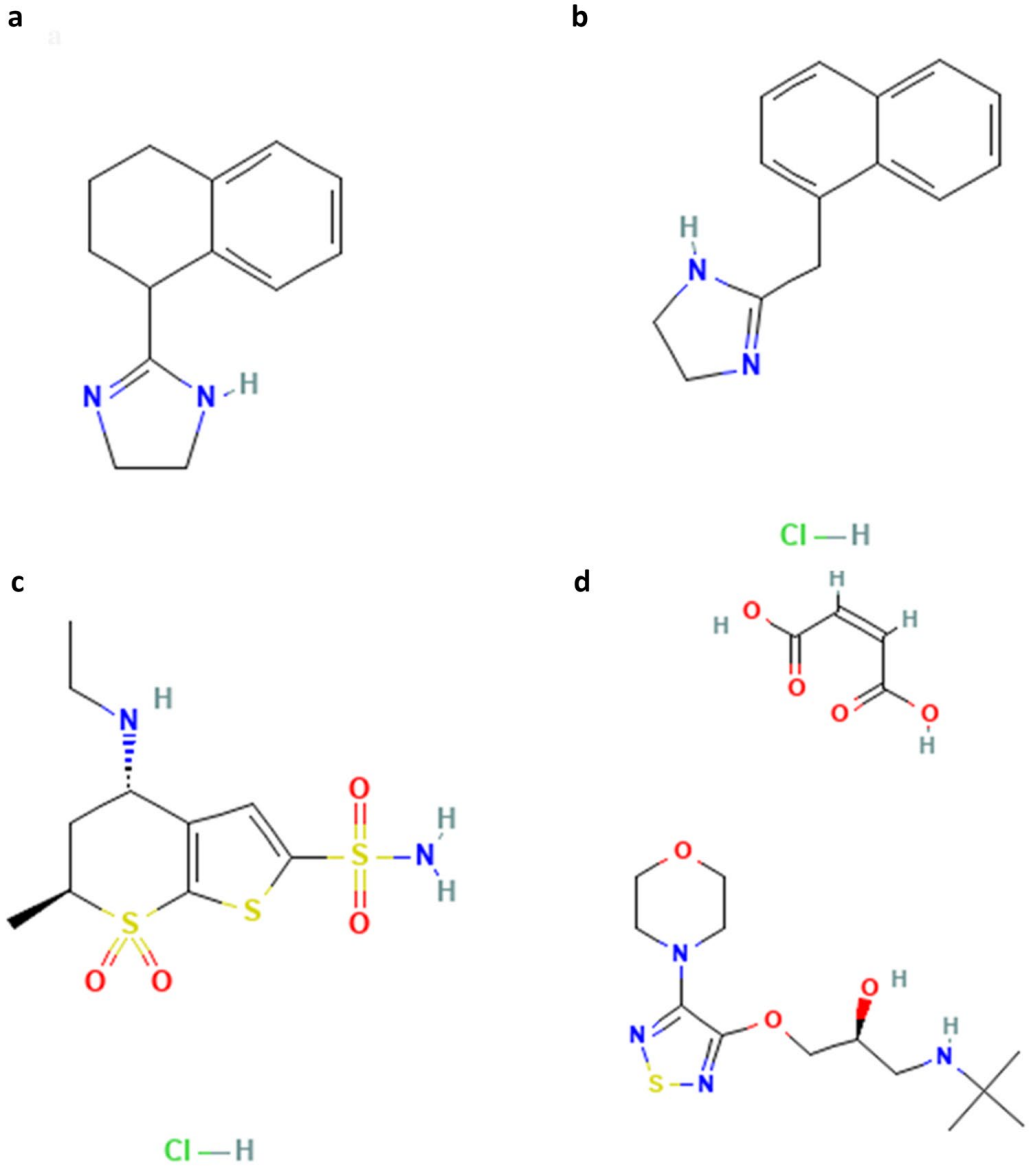
Chemical structures of target molecules demonstrating their free hydrogen bonds which allow for molecular imprinting to occur, (**a**) Tetrahydrozoline^24^, (**b**) Naphazoline^25^, (**c**) Dorzolamide^26^, (**d**) Timolol^27^.

Hydrogels are biocompatible, hydrophilic network polymers with a determinable and tunable stimuli response^8^. Their mechanical and chemical properties have made them of particular interest for use in the biomedical field, especially as sensors, actuators, tissue engineering, and drug delivery devices^9^. Chemical recognition is achieved through interatomic interactions between the target molecules and the functional groups found on the hydrogel backbone. In addition to this recognition, the backbone also allows for insolubility in water^10^. In addition to the backbone, crosslinks allow effective dimensionality to the material which also attributes to hydrogel insolubility in water. Crosslinking can be accomplished through physical or chemical, each of which allow their own benefits whilst presenting unique challenges^11,12^. Physical crosslinks are formed through non-covalent bonds between polar groups on the chain whilst chemical crosslinks are covalent bonds between functional groups on the chain^13^. Between the target molecule and the hydrogel, hydrogen bonds are found. These bonds present a unique balance between strength and responsivity. While other bonds such as covalent or van der Waals interactions exist, these are often poor for a drug delivery system due to their poor balance between strength and responsivity^14^. Through the addition of crosslinkers, a heightened geometry is accomplished which makes a molecular imprinted hydrogel system more plausible^15^. Molecular imprinting, or MIP, is the process in which a target molecule such as a drug or analyte is mixed with a pre-polymerized solution to make a synthetic bonding system which allows for the hydrogel to recognize the target molecule and form covalent or non-covalent bonding^16,17^. As the solution polymerizes, the target molecule is encapsulated within the mesh of the hydrogel^18^. As covalent bonding is limited in its releasability, it is often desired to use hydrogen bonds to link the crosslinker and target molecule. This allows for the best combination of releasability, stability, and biocompatibility^19^. As hydrogen bonding is the best mechanism allowing for the controlled, sustained release of drug from a hydrogel matrix, the backbone selection was catered to ensure it was achievable with hydrophilic drugs. The two backbones chosen were 2-hydroxyethyl methacrylate and 2-hydroxypropyl methacrylate which both contain necessary functional groups (–OH), which enable hydrogen bonding with the target molecules in question^20^. To address the pH effect during glaucoma conditions in the eye, a pH-sensitive monomer was chosen with a tertiary amine group (–N–2(CH_3_)), 2-(dimethylamino) ethyl methacrylate, which becomes protonated at a pK_a_ near 7.0^21^. Under these conditions, interactions with negatively charged ions in the media solution are possible. These interactions cause electrostatic repulsion in the backbone itself, thus causing swelling. During this hydrogel swelling, deformation of the pockets containing target molecules occurs which drastically decreases the thermodynamic stability of these pockets, facilitating release of target molecule from the matrix entirely^22,23^. Tetrahydrozoline, naphazoline, dorzolamide, and timolol maleate were selected as the target molecules of interest due to their ability to form hydrogen bonds with the hydrogel matrix and the varied molecular weights to allow for understanding of the relationship between the hydrogel and the target molecule molecular weights. The four target molecules molecular structure are represented below in Fig. 1.

In the current state of the art, release mechanisms are well-established through literature, where these take the form of diffusion, swelling, or chemical-control mechanisms^28^. The most common for hydrogels is the diffusion-controlled mechanism, in which hydrogels release target molecule following Fickian diffusion^29^. Beyond this, however, there are some considerable knowledge gaps when hydrogels are analyzed through a structure–property relationship lens, notably the influences of hydrogel molecular weight and drug molecular weight. The work presented here seeks to explore the relationship between methacrylate hydrogels and the molecular weight of both drug molecules and the hydrogel. Beyond this, it is also desired to understand the long-term capability of hydrogels to uptake and release drug, as there are some concerns with the degradation response of hydrogels over long periods of time^30^.

## Materials and methods

Ammonium persulfate (APS), 2-(dimethylamino) ethyl methacrylate (DMAEMA), ethylene glycol (EG), 2-hydroxyethyl methacrylate (HEMA), 2-hydroxypropyl methacrylate (HPMA), ethylene glycol dimethacrylate (EGDMA), polyethylene glycol dimethacrylate (PEGDMA), tetraethylene glycol dimethacrylate (TEGDMA), N,N,N’,N’-tetramethylethylenediamine (TEMED), tetrahydrozoline, naphazoline, Timolol maleate, and dorzolamide hydrochloride were purchased from Sigma-Aldrich (St. Louis, MO, US) and used as received for synthesis.

Hydrogels were synthesized through thermally initiated, free radical polymerization which began by preparing two precursor solutions, denoted solution A and solution B. Solution A was prepared by mixing the free radical initiator (APS) and a solvent (EG) for 10 min at room temperature allowing for complete absorption of the APS into the EG. Upon preparation, solution A was set aside to be mixed with solution B. Solution B was prepared by mixing the backbone (HEMA or HPMA), a pH-sensitive monomer (DMAEMA), crosslinker (EGDMA, PEGDMA, or TEGDMA), a catalyst (TEMED), and target molecule (Tetrahydrozoline, Naphazoline, Dorzolamide, or Timolol maleate) for 10 min at room temperature allowing for complete mixing of the solution. Once prepared, solution B was allowed to imprint for 24 h at 5.0 °C. Upon this 24 h imprinting time, solution B was combined into solution A, mixed for 10 min at room temperature and then poured into a mold. From here, the mold was covered and placed in the fridge for 24 h at 5.0 °C. On the next day, the sample could be sufficiently prepared as needed for experimentation. For all experiments mentioned herein, the samples were cut down from their original size into 5 × 5 mm^2^ squares (1.5 mm in depth from the mold) to allow for consistency in dimensionality during testing.

For this experiment a series of six formulations were derived by varying the crosslinker and backbone molecules present in the hydrogels. Amounts of initiator, solvent, backbone, crosslinker, pH-sensitive monomer, and catalyst remained the same throughout the duration of the experiment. The specific compositions are described in Table 1, below.

**Table 1:** Compositions studied in this experiment. Molecular weights are taken from CAS information of the chemical.

Composition	Backbone	Backbone molecular weight (g/mol)	Crosslinker	Crosslinker molecular weight (g/mol)
R1	HEMA	130.14	EGDMA	198.2
R2	HEMA	130.14	TEGDMA	286.32
R3	HEMA	130.14	PEGDMA	400.01
R4	HPMA	144.17	EGDMA	198.2
R5	HPMA	144.17	TEGDMA	286.32
R6	HPMA	144.17	PEGDMA	400.01

The amount of all backbones considered in the experimentation is 68.4% of the total molar-weight.

Crosslinker amount was retained to be 2.44% of the total molar-weight of the system.

The drug was modified a total of four times, denoted by A, B, C, and D annotations on the composition number which proceed alphabetically based on the molecular weight of the drug molecule (A = Tetrahydrozoline (MW = 236.74 g/mol, B = naphazoline (MW = 246.73 g/mol, C = dorzolamide (MW = 324.44 g/mol), D = timolol (MW = 432.51 g/mol))^24–27^. These annotations are added onto the composition such as in R1A, meaning HEMA-EGDMA with a target molecule of tetrahydrozoline, for example. All solutions of drug were prepared using the predominant prescription concentration of solutions, beginning with the raw powder, and mixing with the correct amount of DI water to match. These solutions are then sealed, covered with aluminum foil, and placed into a fridge at 5.0 °C. Tetrahydrozoline, at 0.05%, started with 1.5 mg of powder which was mixed with 30 mL of DI water. Naphazoline, at 0.1%, started with 3.0 mg of powder which was mixed with 30 mL of DI water. Timolol preparation was the same as tetrahydrozoline. Dorzolamide, at 2.0%, started with 6.0 mg of powder mixed with 30 mL of DI water.

A ThermoFisher Scientific (Waltham, MA, USA) Nicolet iS50 Fourier-transform infrared spectrometer (FTIR) was utilized in the study to identify functional groups present in synthesized samples. Absorbance measurements were conducted in the attenuated total reflection (ATR) configuration with a KBR crystal. This setup produced peaks between 1900 and 2400 cm^−1^ and at 3400 cm^−1^ which are not attributed to known functional groups present in the synthesized polymers.

Using a PerkinElmer (Waltham, MA, USA) Lambda 950 UV–Vis–NIR spectrophotometer, transmittance of samples was studied to determine the optical properties of the prepared hydrogels. In addition to the optical transmittance of the samples themselves, UV–Vis was utilized to determine the absorbance of drug solution to determine the drug released.

Drug release experiments were conducted using phosphate buffered saline solution (PBS) with a pH of 7.4. Samples were completely submerged in the solution, allowing for drug release in the x, y, and z-directions. Drug was allowed to release for 12 h at intervals of 1 h, where the drug was released in one media solution, and then submerged into a second solution for 24 h to release the remainder of the drug. Both solutions were characterized to determine the concentration of drug released into each solution and to determine the percentage of drug released at each interval. The drug release experiment was expanded as needed to test the durability of the hydrogel over a months’ time, for instance.

Absorbance data from spectrophotometry was utilized to determine the concentration of target molecule through the Beer-Lambert Law. Herein, A is the absorbance, ε is the molar absorption coefficient, l is the path length, and c is the solution concentration. This formula states that the absorbance of solution is directly proportionate to the concentration when assumed that the molar absorption coefficient and path length remain constant. The Beer-Lambert Law is stated below in Eq. (1).

1 $${\text{A}}\, = \,\varepsilon {\text{lc}}{.}$$

## Results

### Target molecule concentration standards

Through varying the concentration of target molecule from 0.005 mM to 0.10 mM at the peak signature of the target molecule, being 235 nm for tetrahydrozoline, 300 nm for naphazoline, 250 nm for dorzolamide, and 295 nm for timolol. Knowing the concentration of target molecule present in the mixture along with the peak UV–Vis–NIR signature, allows development of a calibration curve which can be used to back solve for the concentration present in a drug kinetics and release experiment. From here, target molecule calibration curves were obtained (Table 2).

**Table 2:** Each drug tested through the course of this study with the corresponding target molecule concentration equation shown. The equations provided within the table represent Eqs (2)-(5) as referenced within the text from hereon.

Drug	Equation
Tetrahydrozoline	(2) $\text{y(x)}=0.4512\text{x}+0.3215$
Naphazoline	(3) $\text{y(x)}=0.3305\text{x}+1.4777$
Dorzolamide	(4) $\text{y(x)}=0.3911\text{x}+0.9012$
Timolol	(5) $\text{y(x)}=7.9878\text{x}+0.4068$

Each drug was tested using the same range of concentrations with the peak signature varying for each. These concentrations are developed from this information.

The linear equations in Eqs. 02, 03, 04, and 05 exhibit comparable linear behavior to the Beer-Lambert Law of Eq. (1), which makes this a confident model for determining the concentration of target molecules present in the samples. The equation y-term expresses the absorbance and x represents the concentration of target molecule in mM. These were repeated a course of seven times per target molecule to allow for confirmation of repeatability.

### Chemical composition analysis

**Figure 2:**
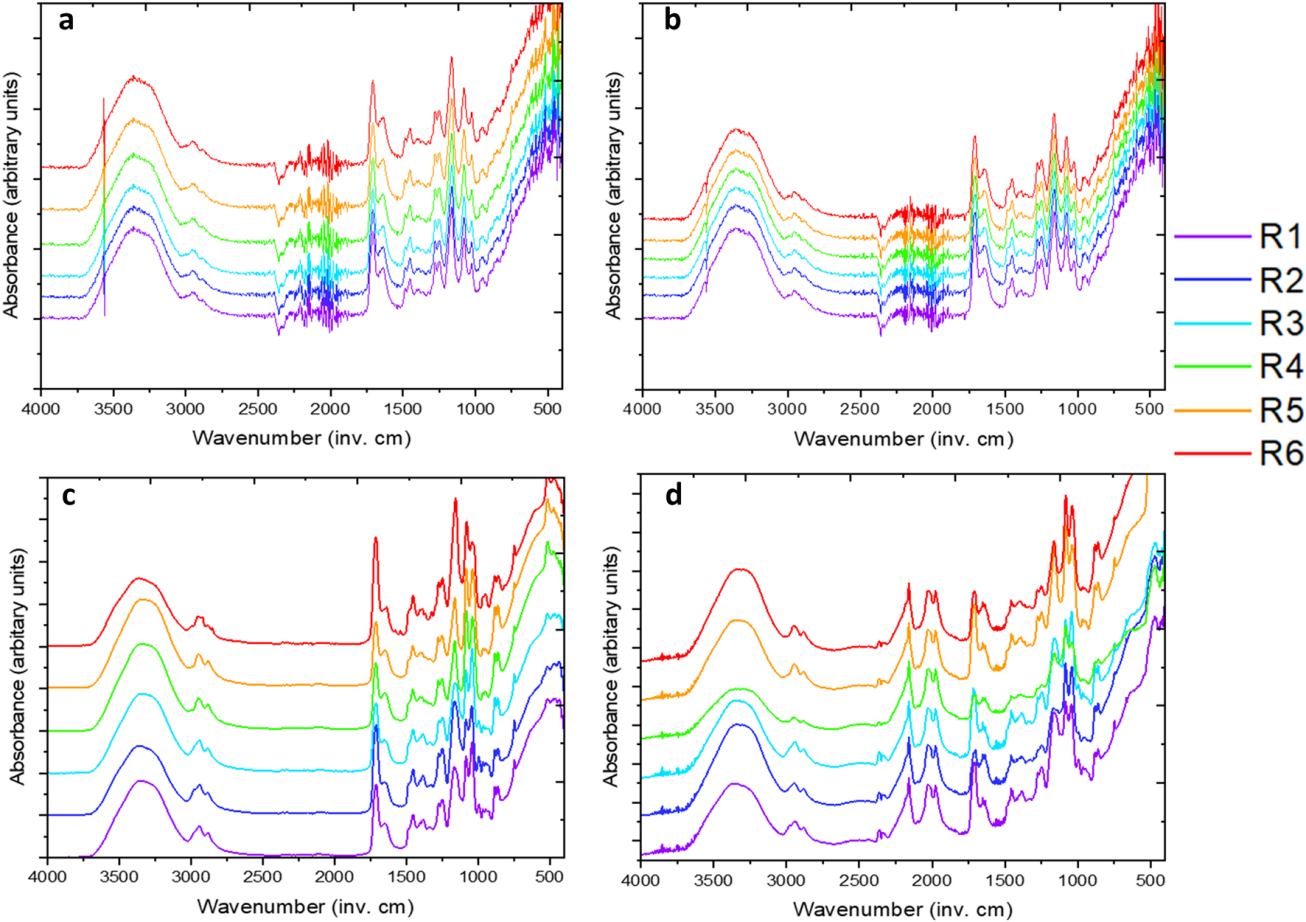
FTIR spectrum for the six compositions. X-axis represents the wavenumber (inv. cm) while the y-axis represents the absorbance (arb. Units) but is relative as the samples are stacked onto one another for easier appreciation of the dataset. (**a**) Tetrahydrozoline 0.05%, (**b**) Naphazoline 0.1%, (**c**) Dorzolamide 2.0%, (**d**) Timolol 0.05%.

It was desired to understand the relative chemical composition, and more specifically, the key functional groups present in the hydrogel material. This serves as both a confirmation that certain groups expected are indeed present and as a measure of any contaminants potentially in the hydrogel. These can be found in Fig. 2, below, with a key subset on the right-side of the figures. From this, it can be noted that the lowest molecular weight is found at the bottom of the FTIR spectrum whilst the highest molecular weight is found at the top of the FTIR spectrum.

FTIR gathered above is valuable in identifying key functional groups present in the samples, particularly as an assurance of composition of the samples. There are noticeable absorption bands of alkene (C=C) at 1505 wavenumber, amine (C–N) at 1317 wavenumber, ether (C–O–C) at 1250 wavenumber, and alkane (C–H) at 659 wavenumber respectively^31^. This corroborates the presumptions of the composition as methacrylate is the dominant structure in the polymer. Note that the intensity of the A and B peaks are lower than C and D peaks, hence the more prominent noise and inclusions from the FTIR-ATR setup. As the absorbance measured increases, this noise decreases relative to the peaks surrounding it.

### Optical property analysis

**Figure 3:**
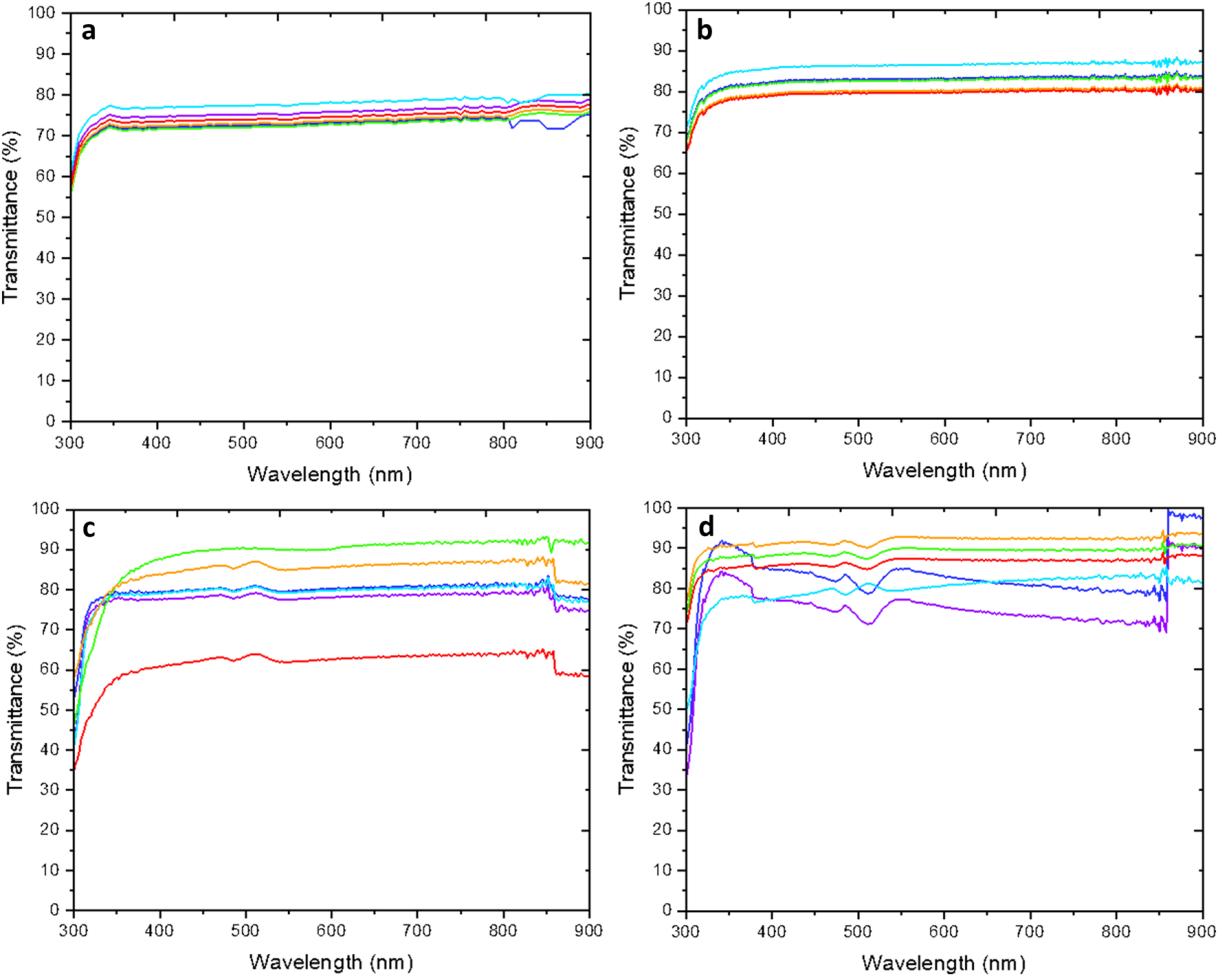
UV–Vis-NIR spectra for all samples studied. Y-axis represents the percent transmittance while the x-axis represents the wavelength, in nm. (**a**) Tetrahydrozoline 0.05%, (**b**) Naphazoline 0.1%, (**c**) Dorzolamide 2.0%, (**d**) Timolol 0.05%.

For ophthalmic drug delivery, it is imperative to determine the influences on the transmission of light that different compositions of target molecule and backbone may have on this property. These results are found in Fig. 3, below.

Through optical investigation of the material, it is clear to determine the performance of properties here which can help further inferences about the capability of certain gels to make useful drug release platforms as a contact lens. It is noticed that the smaller molecular weight drugs offer poorer performance optically than higher molecular weight drugs. Timolol, at least according to this perspective, hinders the optical capability of the hydrogel the least, with near or over 90% transmittances seen.

### Target molecule release kinetics

**Figure 4:**
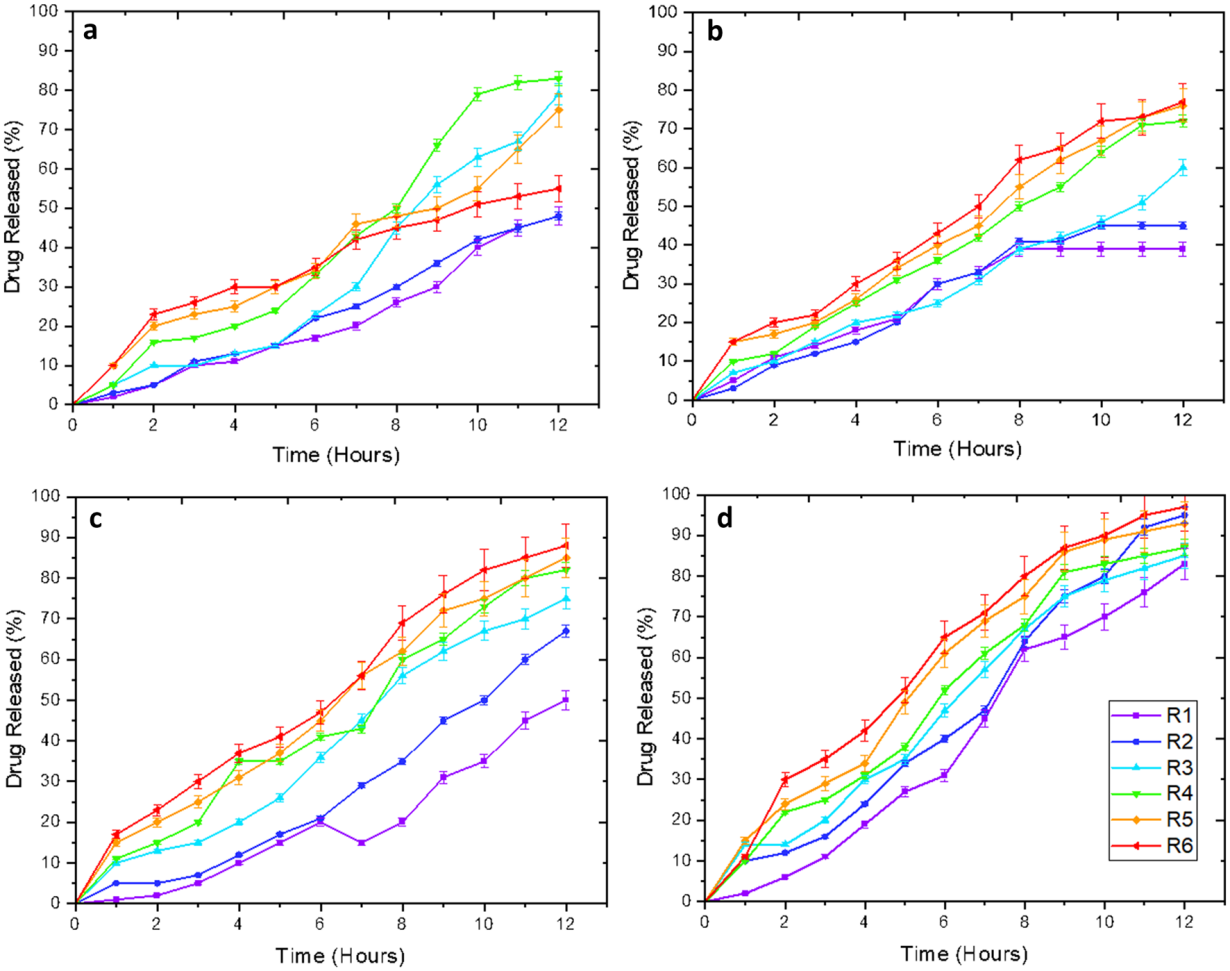
Demonstrating the drug release concentrations of compositions with varying molecular weight. The concentration of target molecule, in mM, is shown on the y-axis. The x-axis demonstrates the time, in hours, of measurement. (**a**) Tetrahydrozoline 0.05%, (**b**) Naphazoline 0.1%, (**c**) Dorzolamide 2.0%, (**d**) Timolol 0.05%.

Through the investigation of the drug release capability over a 12-h period, it is possible to determine how this hydrogel system would function realistically as a contact lens. Measurements made every hour allow for tracking the hydrogels release throughout this period, which, when combined with a standardization of hydrogel size, can be used to compare with the other hydrogel constructions made. Higher molecular weights tend to release quicker than the lower molecular weight hydrogels in most cases. This generalized trend suffers when considering higher molecular weight drugs, which transmit from the matrix equally in high and low molecular weight hydrogel constructions. Figure 4, below, demonstrates the release profile of the target molecule from the hydrogels.

**Figure 5:**
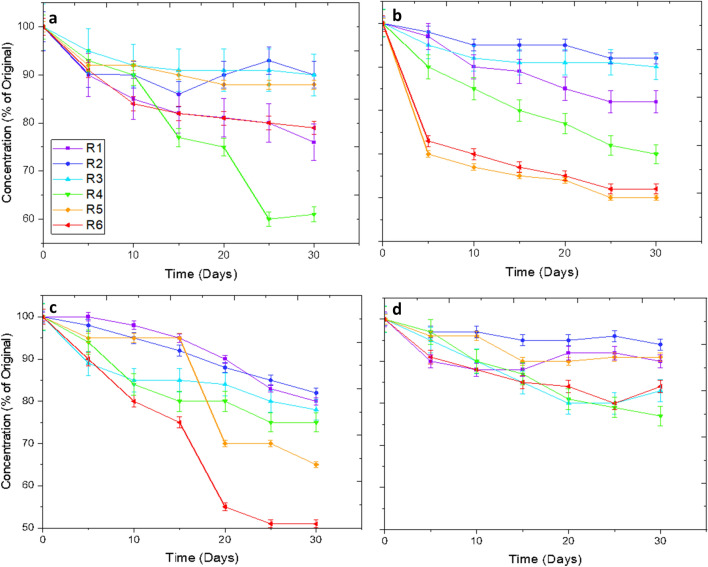
Showing the stability of drug concentration over the course of 30-days for each of the compositions with varying molecular weight and drug. The concentration of target molecule, relative to original loading is shown, in %, on the -axis. The x-axis demonstrates the time, in days, of measurement. (**a**) Tetrahydrozoline 0.05%, (**b**) Naphazoline 0.1%, (**c**) Dorzolamide 2.0%, (**d**) Timolol 0.05%.

Envisioning the release from a hydrogel over a 12-h period is important and allows for important inferences as to how long a drug will take to transmit from a hydrogel yet does not offer sufficient understanding into long-term stability of the hydrogel material. An analysis of a 30-day stability of loading concentration in these hydrogels allows for a more appropriate understanding. This can be seen in Fig. 5, below.

In this figure above, high molecular weight hydrogels tend to suffer with more inherent instability of their matrix, whilst lower molecular weight hydrogels tend to have longer stability when put through 60 uptake-release cycles over the course of 30-days. Timolol, the highest molecular weight drug, has the best stability long term with only a 20% loss in performance of the medium molecular weight hydrogels whilst retaining a range of 85–95% loading capacity through the 30-day consideration. This is compared with other trials such as tetrahydrozoline and dorzolamide which lose almost 50% of their loading capacity with some hydrogels towards the end of the 30-day period.

## Discussion

FTIR measurements conducted, shown in Fig. 2, demonstrate the functional groups present in the synthesized polymers, allowing for a double check as to the expected end-result properties of synthesized hydrogels. In this, the feed composition and result composition corroborate one another as the hydrogel is determined to be alkene-containing due to the presence of acrylates added into the system through the backbone, crosslinker and pH-sensitive monomer components. Variances in the spectra are thus attributable to the target molecule themselves, yet for the most part, the variances in the spectra are minimal and thus allow for easy comparison to all four target molecule containments.

Figure 3 demonstrates the optical properties of the hydrogels, in which it can be noted that there is a minor dependence on the molecular weight of the drug and of the hydrogel with relation to transmittance. Throughout the tetrahydrozoline trial, the transmittance of the hydrogel stays within the 68–76% regime, with lower molecular weight hydrogels showcasing a higher transmittance. This is found as well during the naphazoline trial as well, with the average transmittance being in the 78–85% regime, with again, lower molecular weight hydrogels demonstrating a higher transmittance when compared with their higher molecular weight counterparts. This general trend deteriorates however when the dorzolamide and timolol trials are investigated. It is here that ranges of 55–90% and 69–93%, respectively, are found, without the dependence on the molecular weight of the hydrogel as previously found in the tetrahydrozoline and naphazoline studies. All trials decay their capability to perform in the sub-300 nm region, with tetrahydrozoline decaying at around 295 nm and timolol decaying at around 275 nm. It is from here that one can determine the higher molecular weight drugs allow for a heightened transmittance of the overall hydrogel because of filling the pores better. In the timolol case with lower molecular weight hydrogels suffering to perform as well optically as their higher molecular weight counterparts, it is determinable that the pore size allowed by these hydrogels are not enough to contain the timolol molecule completely, thus resulting in timolol residing on the surface of the hydrogel as opposed to being contained within. This is evidenced by a significant deterioration of the optical properties compared with the higher molecular weight hydrogels.

Through Figs. 4 and 5, it is allowed for the study of how the molecular weight of target molecule and hydrogel play a role in the end-result capabilities of the hydrogel. Through Fig. 4, the release across 12-h is noted. Herein, all hydrogel constructions seem to be capable of this 12-h release profile, through with higher molecular weight hydrogels often having an easier ability to release the drug. Of course, with the lower molecular weight drug constructions, this overall trend begins to deteriorate, with the lower molecular weight hydrogels being able to deliver much better comparably. This is likely due to an optimal relationship between the molecular weights of the drug and the hydrogel, respectively. This said, peak release percentages are 83% for tetrahydrozoline, 76% for naphazoline, 87% for dorzolamide, and 96% for timolol, which demonstrates that higher molecular weight drugs are more easily removed from the hydrogel. This is in part due to the strength in stimuli-responsive capability of these higher-order drugs alongside the increased void size created in the hydrogel.

When considering Fig. 5, there is a distinct relationship between the drug molecular weight, the hydrogel molecular weight, and the stability over 30-days of these constructions. As the drug molecular weight increases, there is an increase in the stability of the absorption–desorption relationship whereas by increasing the hydrogel molecular weight, there is a decrease in this relationship. Overall, the tetrahydrozoline and dorzolamide samples suffer the most depreciation in drug absorption capability, with near 50% losses in the volume able to be absorbed by these constructions at day 30 whilst the naphazoline and timolol lose 35% and 25% maximum, respectively. Of course, this short-term loss in concentration is of concern, yet it provides invaluable information as to how hydrogels can be cycled repeatedly to determine if there is a limit to the recyclability and when the hydrogels would need to be hypothetically “changed” akin to a contact lens.

Future investigation into the hydrogel would be the determination of an agent to allow for prolonged periods of drug release, much longer than 30-days prior to being changed. In addition, a further understanding of the structure–property relationships needs to be ironed out. Specifically, there needs to be further understanding of how molecular weight directly affects the surface of the hydrogel to ensure if there is any long-term loss in the functionality of the hydrogel.

## Conclusions

In this paper, it was demonstrated how the molecular weight of the hydrogel itself and the target molecule contained within influence the end-result properties of the hydrogel, notably the drug release capability and the stability of the drug release. Through varying these two components, a structure–property relationship was established, allowing for future research scopes into the structure of a hydrogel.

## Data Availability

Data can be made available from the authors upon reasonable request. Please contact the corresponding author, Jeffrey Bates for data requests.
